# Homogalacturonan deesterification during pollen–ovule interaction in *Larix decidua* Mill.: an immunocytochemical study

**DOI:** 10.1007/s00425-014-2074-6

**Published:** 2014-05-04

**Authors:** Katarzyna Rafińska, Michał Świdziński, Elżbieta Bednarska-Kozakiewicz

**Affiliations:** Department of Cell Biology, Faculty of Biology and Environment Protection, Nicolaus Copernicus University, Lwowska 1, 87-100 Toruń, Poland

**Keywords:** Adhesion, Extracellular matrix, Gymnosperms, Pollen grain, Pollen tube growth, Pollination

## Abstract

Studies on angiosperm plants have shown that homogalacturonan present in the extracellular matrix of pistils plays an important role in the interaction with the male gametophyte. However, in gymnosperms, knowledge on the participation of HG in the pollen–ovule interaction is limited, and only a few studies on male gametophytes have been reported. Thus, the aim of this study was to determine the distribution of HG in male gametophytes and ovules during their interaction in *Larix decidua* Mill. The distribution of HG in pollen grains and unpollinated and pollinated ovules was investigated by immunofluorescence techniques using monoclonal antibodies that recognise high methyl-esterified HG (JIM7), low methyl-esterified HG (JIM5) and calcium cross-linked HG (2F4). All studied categories of HG were detected in the ovule. Highly methyl-esterified HG was present in the cell walls of all cells throughout the interaction; however, the distribution of low methyl-esterified and calcium cross-linked HG changed during the course of interaction. Both of these categories of HG appeared only in the apoplast and the extracellular matrix of the ovule tissues, which interact with the male gametophyte. This finding suggests that in *L. decidua,* low methyl-esterified and calcium cross-linked HG play an important role in pollen–ovule interaction. The last category of HG is most likely involved in adhesion between the pollen and the ovule and might provide an optimal calcium environment for pollen grain germination and pollen tube growth.

## Introduction

In seed plants, the extracellular matrix of female reproductive organs is where interaction between ovules and the male gametophyte occurs. In angiosperms, pollen grains germinate on the stigma, and the pollen tube continues its growth towards the female gametophyte in the ecm of the style. In gymnosperm plants, unlike in flowering plants, pollen grains land directly on the ovules; therefore, the male gametophyte interacts only with the ecm secreted by the ovule tissues. However, in both angiosperms and gymnosperms, ecm of the female reproductive organs should provide specific ions and molecules necessary for nutrition, attraction and guidance of the male gametophyte. Many studies in angiosperms have shown that the pollen–pistil interaction involves such ecm components as homogalacturonan, arabinogalactan proteins and calcium ions (Cheung et al. 1995; Jauh and Lord 1996; Mollet et al. 2000; Lenartowska et al. 2001; Zhao et al. 2004; Bednarska et al. 2005; Hristova et al. 2005; Coimbra et al. 2007; Ge et al. 2009; Sage et al. 2009; Costa et al. 2013).

In angiosperms, Ca^2+^ plays a particularly important role in pollen grain germination and pollen tube growth. These ions are taken up by germinating pollen grains and growing pollen tubes forming a specific tip-to-base Ca^2+^ gradient. Elevated levels of Ca^2+^ at the tube tip are involved in vesicle secretion and in determining the orientation of the tube growth (see the review by Ge et al. 2007; Hepler and Winship 2010). Similarly, in gymnosperms, Ca^2+^ is taken up by growing pollen tubes (Lazzaro et al. 2005; Chen et al. 2009), and as Wu et al. (2008) found, the Ca^2+^ influx in *Pinus bungeana* is even higher than in angiosperm pollen tubes. The main Ca^2+^ store in the ecm of plant cells is HG, which is the most abundant pectic polysaccharide (see the review by Wolf et al. 2009). HG is synthesised and methyl-esterified in the Golgi apparatus.

Within the cell wall, high methyl-esterified HG can undergo deesterification by PMEs. These enzymes remove the methyl groups from the HG chain leading to the formation of free carboxyl groups and to the release of methanol and protons. Free carboxyl groups can bind Ca^2+^, and a stretch of at least nine deesterified galacturonic acid residues can form an ‘egg-box’ structure due to the formation of Ca^2+^ cross-bridges. The ‘egg-box’ structures participate in gel formation and, thus, strengthen the cell wall; they can also become a target for pectin-hydrolysing enzymes, such as polygalacturonases and pectin/pectate lyases (see the review by Wolf et al. 2009). The action of PMEs is influenced by a range of factors, including cell wall pH and the pattern of methyl-esterification of HG chains.

Deesterification of HG is a process that plays a significant role in the pollen–pistil interaction in angiosperms. It has been shown that changes in HG methyl-esterification status during the pollen–pistil interaction depend on the type of pistil. In the unpollinated pistil of *Haemanthus albiflos* (dry stigma and hollow style), the high methyl-esterified HG form was mainly detected (Bednarska et al. 2005; Lenartowska et al. 2011); HG deesterification occurs in the cell walls of the stigma and style during pollen germination and pollen tube growth. In *Petunia hybrida* and *Olea europaea* L. (wet stigma and solid style), low methyl-esterified HG was already present in the stigma exudates and ecm of the transmitting tissue during pollination (Lenartowska et al. 2001; Bednarska et al. 2005; Suárez et al. 2013). Additionally, previous studies have indicated that in the transmitting tissue of the *P. hybrida* pollinated style, lysis of deesterified HG was accompanied by a strong increase in Ca^2+^ levels in the ecm (Bednarska et al. 2005). Therefore, in the *P. hybrida*, low-esterified HG can serve as a Ca^2+^ source for pollen tubes growing in the transmitting tract. In the lily, low-esterified HG together with small (9 kDa) proteins is responsible for the in vivo tube adhesion to the wall surface of the stylar epidermis (Mollet et al. 2000; Park et al. 2000).

The role of the components of the ecm in the sexual reproduction of conifers has not been intensively studied. So far, there are only a few reports on this topic, and they focus primarily on pollen tube wall synthesis (Derksen et al. 1999; Mogami et al. 1999; Chen et al. 2008; Wu et al. 2008). To date, there is practically no data on the nature and role of HG during the interaction between the male gametophyte and ovule of gymnosperm plants. Therefore, the aim of this study was to analyse the distribution of different categories of HG in the male gametophyte and ovules of *Larix decidua* before and after pollination. The potential role of HG in the sexual processes of gymnosperms is discussed and includes a comparison with available data on HG behaviour during pollen–pistil interaction in flowering plants.

## Materials and methods

### Plant material

Male and female cones of *Larix decidua* Mill. were collected from trees growing in the garden of the Faculty of Biology and Environmental Protection, Nicolaus Copernicus University, Toruń, Poland.

### Preparation of material

Mature pollen cones were collected in March and April. They were surface sterilised in 70 % ethanol for 40 s and then in 10 % sodium hypochlorite. Cones were rinsed in sterile distilled water and dried at RT in sterile Petri dishes covered with sterile filter paper. Before culturing, pollen grains were hydrated for 24 h at 24 °C in sterile conditions. Subsequently, the prepared pollen was germinated in the medium contained Brewbaker and Kwack minerals diluted 1:10 supplemented with 18 % PG 4000, 7 % sucrose, 0.4 % phytagel, nystatin (0.0041 g/25 ml) and chloramphenicol (0.0014 g/25 ml), and the pH was adjusted to 5.2. In this medium, pollen grains were cultured together with sterilised nucelli and archegonia. The cultivation was carried out at 24 °C in the dark. For immunolocalisation of HG, the pollen tubes were collected after 7 days of growth and fixed in a mixture of 4 % paraformaldehyde and 0.25 % glutaraldehyde in PBS with pH 7.2 for 2 h at RT. The fixed pollen tubes were individually transferred to cover glasses coated with a drop of poly-l-lysine.

**Fig. 1:**
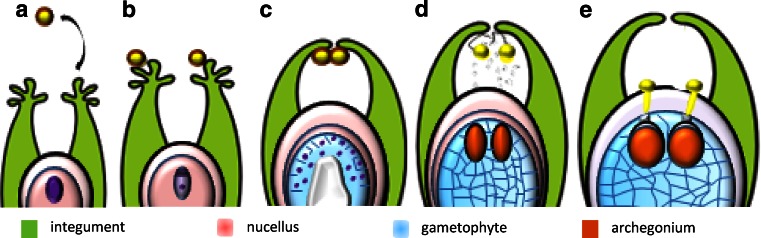
Successive stages of pollen–ovule interaction in *L. decida*. **a** The ovule at the megasporocyte stage—period of the pollen shed. **b** Stage of megaspores—stigmatic tip of the ovule is pollinated. **c** Free nuclear gametophyte stage—pollen grains are engulfed into the micropylar canal of the ovule. **d** Cellular gametophyte stage—pollen grains are carried to the nucellar apex. **e** Mature ovule—pollen tubes penetrate the nucellus

Female cones were collected from March to June. This period encompassed successive stages of the interaction between the male gametophyte and ovule, including pollination, engulfment of pollen grains into the micropylar canal, pollen grain germination and pollen tube growth (Fig. 1). Dissected ovules were fixed in 4 % paraformaldehyde and 0.25 % glutaraldehyde in PBS, placed under vacuum for 1 h and then held at 4 °C overnight. Fixation was followed by buffer rinses (3 times for 20 min). The material was dehydrated in increasing concentrations of ethanol, including several washes in absolute ethanol, and then infiltrated and embedded in BMM resin at −20 °C under UV light for polymerisation (butyl methacrylate, methyl methacrylate, benzoin ethyl ether and dithiothreitol from Sigma-Aldrich). The embedded material was cut on a Leica UCT ultramicrotome into semi-thin serial sections (1,000 nm) and placed on microscope slides coated with biobond (British Biocell International, Cardiff, UK). Before performing immunocytochemical reactions, the BMM resin was removed by washing the slides in pure acetone twice for 10 min. The sections were then washed twice in water and, finally, in PBS.

Pollen grains used for the immunocytochemical reaction were collected from male cones at the time of pollen dehiscence. Before fixation in 4 % paraformaldehyde and 0.25 % glutaraldehyde, the pollen grains were hydrated for 24 h at 24 °C. Fixation was carried out at 4 °C overnight. After three washes in PBS, the pollen grains were embedded in agar blocks by the use of lukewarm 2 % agar. The material was dehydrated, embedded in BMM resin and cut into semi-thin sections as described previously.

### Immunolocalisations

Three different primary antibodies (JIM7, JIM5 and 2F4) (PlantProbes, Paul Knox Cell Wall Lab., University of Leeds, UK) were used for the immunodetection of cell wall antigens. The JIM7 antibody recognises low-unesterified epitopes of HG but does not bind to fully unesterified HG, the JIM5 antibody—fully unesterified and low methyl-esterified HG (Knox et al. 1990; Verhertruggen et al. 2009) and the 2F4 antibody binds specifically to a dimeric association of deesterified HG chains through calcium ions (Liners and van Cutsem 1992). Immunolocalisation of high-esterified and low-esterified HG was performed as previously described by Rafińska and Bednarska (2011). The sections were treated with blocking solution (PBS with 2 % BSA) for 1 h and then incubated with the JIM7 or JIM5 antibody (diluted 1:50 in PBS with 0.2 % BSA) overnight at 4 °C. After washing with PBS, the sections were incubated with secondary rabbit antirat IgG antibody conjugated with Cy3 (Jackson ImmunoResearch Laboratories, West Baltimore Pike, PA, USA) that was diluted 1:100 in PBS with 0.2 % BSA for 1 h in the dark. Finally, the sections were washed with PBS.

To detect calcium cross-linked HG, the sections were blocked with 0.5 % BSA in 20 mM Tris–HCl for 10 min and incubated with primary antibody 2F4 (diluted 1:50 in 0.1 % BSA in 20 mM Tris–HCl) for 1 h at room temperature. Next, the slides were washed in the same buffer four times and incubated with antimouse IgG secondary antibody labelled with Cy3 (Sigma-Aldrich) that was diluted 1:50 in 20 mM Tris–HCl buffer with 0.1 % BSA for 1 h at RT in the dark. Finally, the samples were washed in Tris–HCl.

DNA in the sections was stained with a water solution (4 pg/ml) of DAPI (Fluka) for 5 min. Then, the sections were washed in distilled water, dried at room temperature and covered with 0.5 % *N*-phenylenediamine. Controls were performed omitting the incubation step with the primary antibodies; specific labelling was not observed in all control reactions (cf. Fig. 5j). Observations were performed using a Nikon Eclipse 80i fluorescence microscope. Images were captured by using a Nikon DS-5Mc colour-cooled digital camera paired with the Lucia G 5.30 image analysis software. The camera settings of exposure time, gain and offset were kept constant. Images of in vitro growing pollen tubes were acquired by the confocal Nikon microscope PCM 2000-Eclipse TE 300 paired with the EZ 2000 Viewer software package. The signals from antibodies were observed as a red fluorescence of Cy3, and additional images were collected in the green (autofluorescence) and blue (DAPI fluorescence) channels.

## Results

### Interaction between the male gametophyte and ovule

**Fig. 2:**
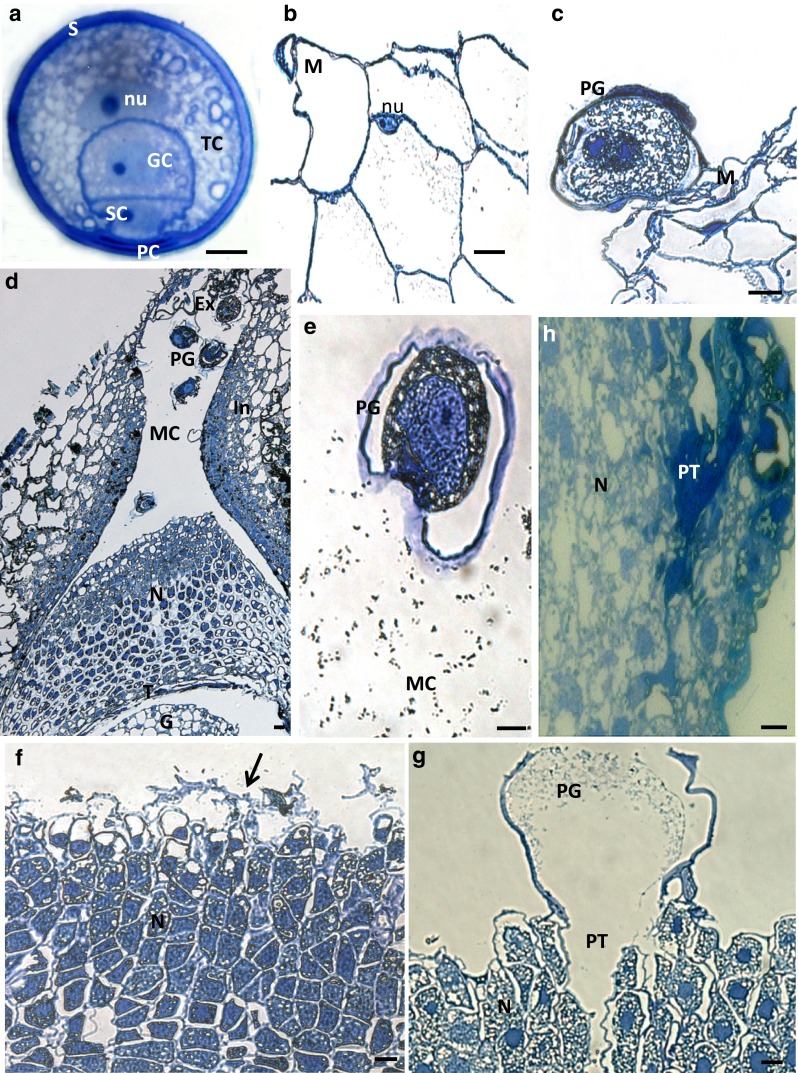
The cytological view of the pollen–ovule interaction in *L. decidua.* **a** A mature pollen grain containing tube cell, generative cell, sterile cell and two degenerated prothallial cell. **b** The strong vacuolated cells of the unpollinated stigmatic tip (stage of megasporocyte). **c** The degenerating cells of the pollinated stigmatic tip (stage of megaspores). **d–f** The ovule at the stage of the cellular gametophyte with a differentiated central cell. **d** Pollen grains in the micropylar canal. At the distal end of the canal, fragments of exine shed by the pollen grain are visible. The cellular gametophyte is surrounded by degenerating tapetal cells. **e** A pollen grain in the micropylar canal filled with secretions. **f** On the surface of the nucellus, secretions (*arrow*) are visible. **g–h** Mature ovule. **g** Pollen grain germinating on the surface of the nucellus. **h** A pollen tube growing in the nucellus; cells surrounding the pollen tube are degenerated. *Ex* exine, *G* gametophyte, *GC* generative cell, *In* integument, *M* stigmatic tip, *MC* micropylar canal, *N* nucellus, *nu* nucleus, *PC* prothallial cell, *PG* pollen grain, *PT* pollen tube, *S* sporoderm, *SC* sterile cell, *T* tapetum, *TC* tube cell. *Scale bars* 10 µm

The successive stages of interaction between the male gametophyte and ovule in larch are schematically shown in Fig. 1 (Rafińska and Bednarska 2011). In *L. decidua,* pollen is shedding when the megasporogenesis takes place in the ovule (Fig. 1a, b). The pollen grains are non-saccate (Fig. 2a) and land on the expanded surface of the integument called the stigmatic tip (Fig. 1b). The cells of the unpollinated stigmatic tip are strongly vacuolated (Fig. 2b) and collapse after pollination (Fig. 2c). Over several days, pollen grains are engulfed into the micropylar canal of the ovule (Fig. 1c). At this time, a functional megaspore develops in the free nuclear gametophyte (Fig. 1c). At the distal end of the micropylar canal, pollen grains hydrate and shed their exine (Fig. 2d). After gametophyte cellularisation (Fig. 1d), fluid secretions fill the canal, and pollen grains, enclosed only by intine, are carried to the nucellar apex (Fig. 2e). At this time, the cells situated in the nucellar apex are smaller and strongly vacuolated, while the cells in the central region of the nucellus are larger and have thicker walls (Fig. 2d). Cells of the apex collapse and secretion appear on the surface of the nucellus (Fig. 2f). Pollen grains germinate on the nucellar surface (Fig. 2g) when the prothallium contains mature archegonia (Fig. 1e). Within few days, the pollen tubes penetrate the nucellus (Fig. 2h), followed by the neck cells and ventral canal cell, and grow into the egg cell.

### Localisation of HG epitopes in the mature pollen grain and unpollinated ovule

**Fig. 3:**
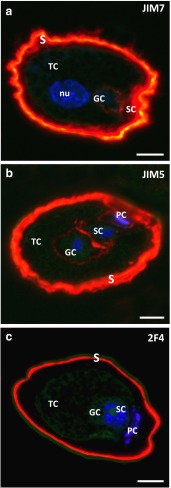
Immunolocalisation of HG in the mature larch pollen grain. **a** An intense JIM7 labelling was present in the pollen wall. Lower fluorescence was visible in the walls of pollen cells. **b** JIM5 labelled the wall of the pollen grain as well as pollen cells. **c** 2F4 labelling was found only in the pollen wall. *GC* generative cell, *nu* nucleus, *PC* prothallial cell, *PG* pollen grain, *S* pollen wall, *SC* sterile cell, *TC* tube cell. *Scale bars* 10 µm

In the wall of the mature, hydrated pollen grain, HG epitopes recognised by JIM7 (Fig. 3a), JIM5 (Fig. 3b) and 2F4 (Fig. 3c) Abs were observed. Weak fluorescence from JIM7 and JIM5 antibodies was also present in the walls of the generative and sterile cells as well as between the prothallial and sterile cells (Fig. 3a, b). In the pollen cell walls, labelling with 2F4 was not detected (Fig. 3c).

**Fig. 4:**
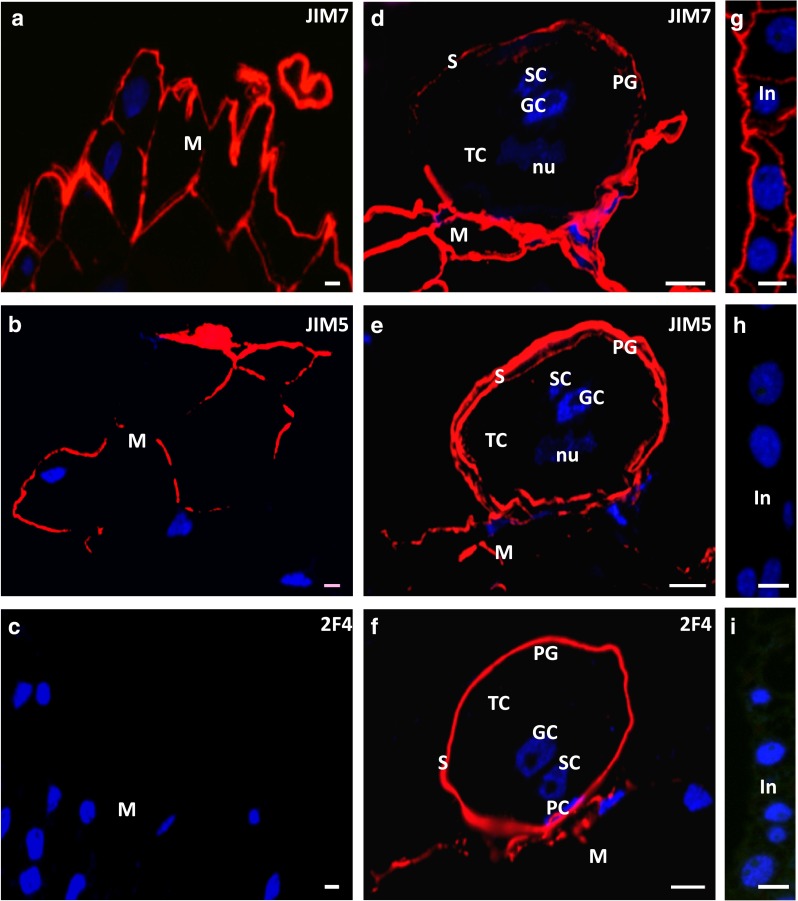
Immunolocalisation of HG in the unpollinated and pollinated larch ovule. **a–c** The unpollinated stigmatic tip. **a** JIM7 labelling was seen in the walls of all cells**. b** JIM5 labelling was present only in some walls. **c** 2F4 labelling was not detected. **d–f** The pollinated stigmatic tip. **d** For the JIM7Ab, strong signal was present in the walls of the degenerating stigmatic tip cells, and a weaker labelling occurred in the pollen grain wall. **e** With regard to the JIM5 Ab, a weak fluorescence was observed in the degenerating stigmatic tip, and a strong labelling in the pollen grain wall. **f** At the place of pollen grain adhesion, on the surface of the stigmatic tip and in the pollen grain wall, the epitope recognised by 2F4 was visible. **g–i** The integument. **g** In the cell walls, signal of JIM7 was found. **h**, **i** The integument was not labelled by JIM5 (**h**) or 2F4 (**i**). *GC* generative cell, *In* integument, *M* stigmatic tip, *PC* prothallial cell, *PG* pollen grain, *S* pollen wall, *SC* sterile cell, *TC* tube cell. *Scale bars* 10 µm

In the ecm of the unpollinated stigmatic tip, the main epitope found was that recognised by the JIM7 Ab (Fig. 4a). In this tissue, the JIM5 fluorescence was irregular and more intense on the stigmatic tip surface (Fig. 4b). Before pollination, fluorescence of the 2F4 Ab was not detected (Fig. 4c).

### Localisation of HG epitopes in the pollinated larch ovule

#### Pollination stage

Pollination induced changes in the distribution of HG epitopes in both the pollen grain and stigmatic tip. In the wall of the pollen grain adhering to the stigmatic tip, the level of fluorescence after labelling with the JIM7 Ab was significantly lower than before pollination (Fig. 4a, d). The strong labelling with the JIM5 Ab was localised to the pollen wall, and the fluorescence was visible as two layers (Fig. 4e). None of the HG epitopes were detected in the thin walls of the pollen cells (Fig. 4d, e). The fluorescence derived from the 2F4 Ab was observed only in the pollen wall (Fig. 4f). After pollination, the epitope recognised by the 2F4 Ab also appeared in the stigmatic tip but only at the place of pollen grain adhesion (Fig. 4f). The walls of the degenerating stigmatic tip cells were still labelled by JIM7 (Fig. 4d) and JIM5 (Fig. 4e) Abs. At this time, only the low-unesterified form of HG was detected in the integument (Fig. 4g–i).

#### Pollen grains in the micropylar canal

**Fig. 5:**
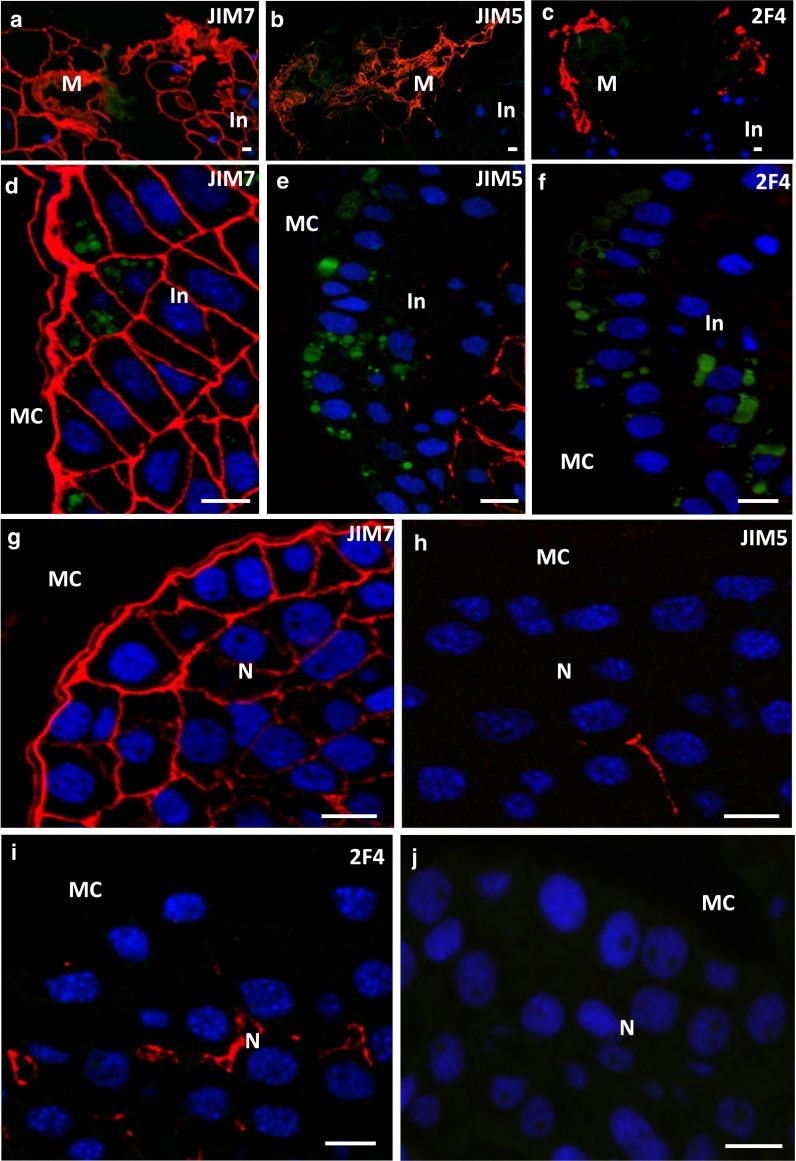
Immunolocalisation of HG in the larch ovule after the engulfment of the pollen grain into the micropylar canal—free nuclear gametophyte stage. **a–c** The stigmatic tip. **a** A JIM7**, b** JIM5, **c** 2F4 labelling of all studied HG epitopes occurred in the material formed as a result of the degeneration of the stigmatic tip cells. **d–f** The integument. **d** JIM7 signal was present in the cell walls of all cells of the integument. **e** JIM5 fluorescence was detected only in the cell walls situated deep within the integument. **f** The integument was completely devoid of fluorescence from 2F4. **g–i** The nucellus. **g** Epitopes recognised by JIM7 were visible in all the cell walls of the nucellar cells. **h**, **i** A JIM5 (**h**) and 2F4 (**i**) were localised only in some cell walls situated below the surface of the nucellus. **j** The negative control showed no labelling in the ovule when the primary rat antibody was omitted. *In* integument, *M* stigmatic tip, *MC* micropylar canal, *N* nucellus. *Scale bars* 10 µm

At the time of pollen grain engulfment into the micropylar canal, all analysed epitopes were detected in the material formed as a result of stigmatic tip degeneration (Fig. 5a–c). The epitope bound by JIM7 was mainly localised to the apoplast of the integument (Fig. 5d). Particularly, intense signal, sometimes observed as two layers, was observed in the integumentary wall coating the micropylar canal. Surface cells of the integument were not labelled with the JIM5 (Fig. 5e) or with 2F4 Abs (Fig. 5f). The epitope recognised by the JIM5 Ab was only detected in the walls of cells situated deep in the integument (Fig. 5e).

**Fig. 6:**
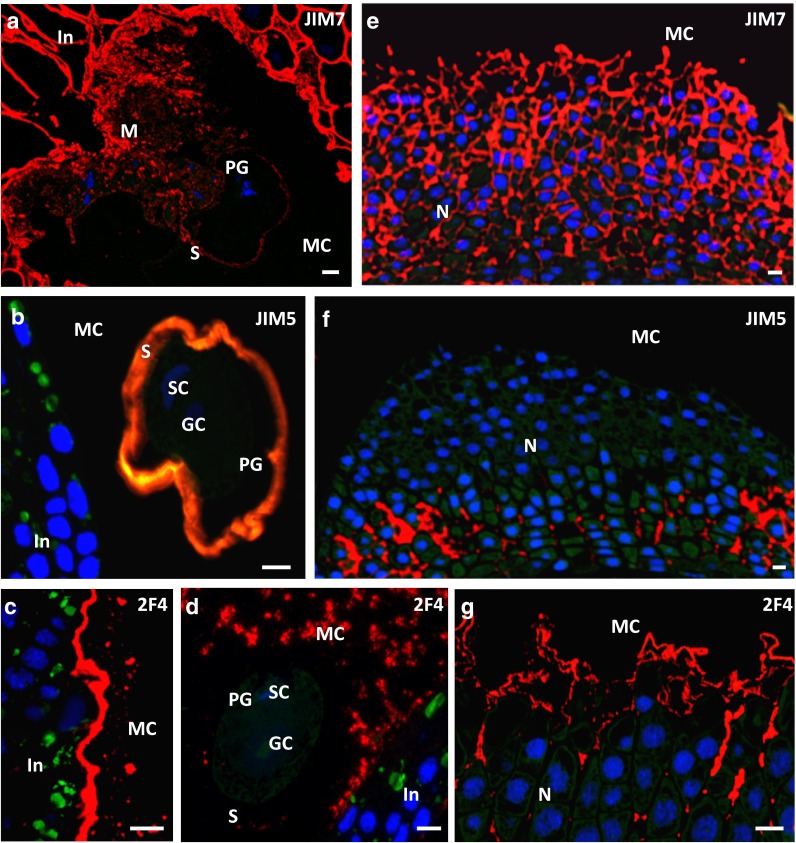
Immunolocalisation of HG in the larch ovule after the engulfment of the pollen grain into the micropylar canal—cellular gametophyte stage. **a–d** The pollen grain in the micropylar canal. **a** The JIM7 fluorescence in the pollen grain wall was weak. Intense fluorescence was visible in the remnants of the degenerated cells of the stigmatic tip and in the walls of the integumentary cells. **b** The JIM5 labelling was localised to the pollen grain wall. The micropylar canal and apoplast of the integument were devoid of fluorescence. **c** The 2F4 signal shortly after engulfment of pollen grains was strong on the surface of canal, and clusters of fluorescence were also visible in the micropylar canal. **d** Later, the apoplast of the integument was not labelled by 2F4. Numerous clusters of fluorescence were observed in the material surrounding the pollen grain. The pollen grain wall was almost completely devoid of the labelling. **e–g** The nucellus. **e** The JIM7 signal was found in all the cell walls and on the nucellar surface. **f** Single clusters of JIM5 fluorescence were observed in the proximal region of the nucellus. **g** The 2F4 labelling was visible in the cell walls of the cells situated in the apical region of the nucellus and in the secretions on its surface. *GC* generative cell, *In* integument, *M* stigmatic tip, *MC* micropylar canal, *N* nucellus, *PG* pollen grain, *S* pollen wall, *SC* sterile cell, *TC* tube cell. *Scale bars* 10 µm

Immediately after the engulfment of pollen grains into the micropylar canal, the first changes in the distribution of the HG epitopes were observed in the nucellus. The walls of the nucellus cells were still labelled by the JIM7 Ab (Fig. 5g); however, for the first time, this tissue showed a weak labelling with the JIM5 (Fig. 5h) and 2F4 Abs (Fig. 5i). During gametophyte cellularisation and archegonia differentiation, the wall of the pollen grain present in the micropylar canal was devoid of exine. In this wall, the JIM7 Ab labelling decreased further (Figs. 6a, 4d), and the JIM5 Ab labelling increased (Figs. 6b, 4e). The fluorescence indicating the presence of calcium cross-linked HG almost completely disappeared (Fig. 6d). In the area of the degenerating stigmatic tip, strong labelling with the JIM7 Ab was observed at the site of pollen grain adhesion and in the cell walls of the integument (Fig. 6a); however, the cell walls of the integument were not labelled by the JIM5 Ab (Fig. 6b).

The 2F4 Ab intensively labelled the apoplast coating the micropylar canal (Fig. 6c). In the ecm of the canal, the signal was observed as small sparse clusters. During ovule development, a decrease in the level of 2F4 Ab labelling occurred in the inner surface of the integument and was accompanied by an increase in the number of fluorescence clusters in the micropylar canal (Fig. 6d). At this time, considerable changes in HG distribution were observed in the nucellus. In the ecm of the nucellus, all examined epitopes were present; however, their distribution was different. JIM7 Ab fluorescence was observed in both, the secretions present on the nucellar surface and in the walls of the epidermal and subepidermal cells (Fig. 6e). Epitope recognised by the JIM5 Ab was not detected in the apoplast of epidermal or subepidermal cells (Fig. 6f). However, the label was present in the ecm of cells situated deeper in the nucellus (Fig. 6f) and was visible as numerous, irregular clusters. Such a localisation pattern was characteristic for this period, as fluorescence was observed almost exclusively in the longitudinal cell walls of the nucellus (Fig. 6f). The epitopes recognised by 2F4 were localised in the walls of the epidermal and subepidermal cells of the nucellus as well as in the secretions present on its surface (Fig. 6g).

### Pollen grain germination and pollen tube growth—the stage of the mature ovule

**Fig. 7:**
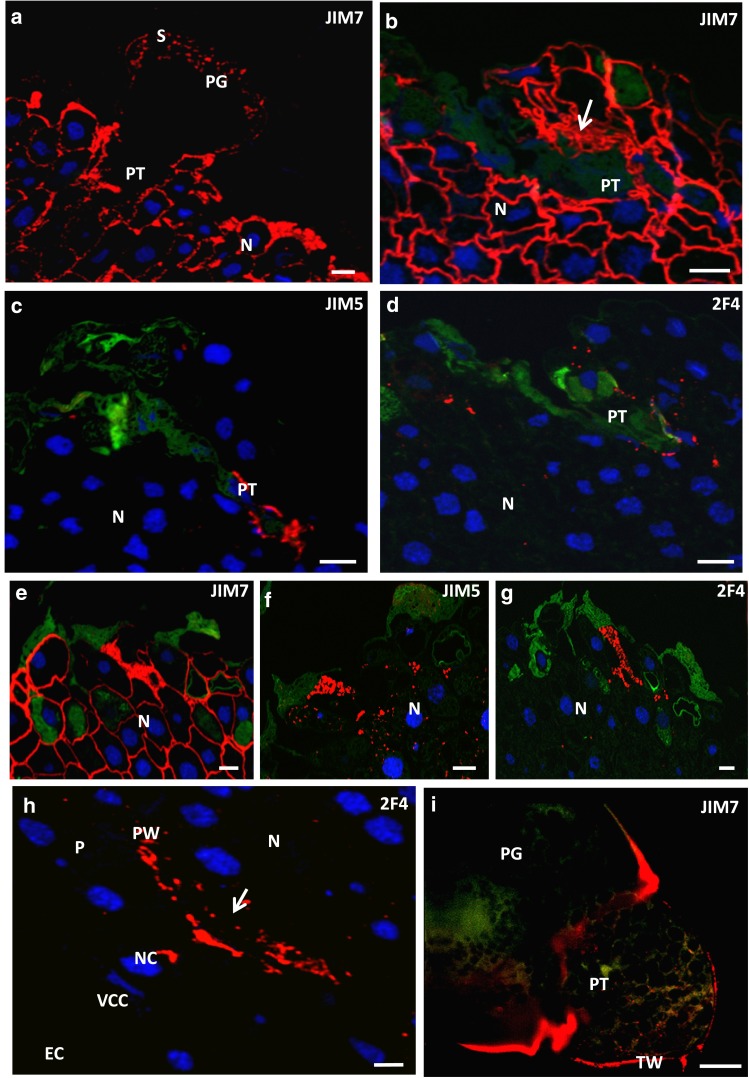
Immunolocalisation of HG in the mature ovule. **a–d** Pollen grain germination and pollen tube growth. **a** The JIM7 labelling was weak in the pollen grain wall and likely also in the pollen tube wall; small clusters of fluorescence were localised in the pollen cytoplasm. Intense labelling was visible in all the cell walls of the nucellar cells. **b–d** The site where the pollen tube overgrows the nucellus. **b** The JIM7 was present in the nucellar cell walls, and intense labelling was visible in the degenerated cells adjacent to the pollen tube (*arrow*). **c** Signal of JIM5 was seen only in the area of pollen tube growth. **d** The 2F4 labelling existed as single spots localised mainly in the area of pollen tube growth. **e–g** The nucellus of the mature ovule, the site where the pollen grain is not present. **e** The fluorescence from JIM7 was present in the cell walls, but labelling was not visible on the nucellar surface. **f**, **g** Signal from JIM5 (**f**) and 2F4 (**g**) was detected only in the apical region of the nucellus. **h** The area between the nucellus and prothallium. The fluorescence from 2F4 was localised only in the wall separating the prothallium from the nucellar cells. **i** Immunolocalisation of JIM7 in the in vitro growing larch pollen tube. The labelling was strong in the intine surrounding the germinating pollen tube. In the pollen tube, the fluorescence was visible in the wall and in small clusters in cytoplasm. *EC* egg cell, *N* nucellus, *NC* neck cell, *nu* nucleus, *P* prothallium, *PG* pollen grain, *PT* pollen tube, *PW* prothallial wall, *S* pollen wall, *TC* tube cell, *VCC* ventral canal cell, *TW* tube wall. *Scale bars* 10 µm

At the time of archegonia maturity, germinating pollen grains and growing pollen tubes were visible on the nucellar surface. In the wall of the germinating pollen grains, a weak fluorescence from the JIM7 Ab was still observed (Fig. 7a). The JIM7 Ab labelling was also present in the pollen cytoplasm.

During pollen germination and pollen tube growth, a strong labelling of JIM7 Ab was still localised in the walls of the nucellar cells (Fig. 7a, b) as well as in the area of pollen tube growth (Fig. 7b). Epitopes recognised by JIM5 (Fig. 7c) and 2F4 Abs (Fig. 7d) were also detected in the area of pollen tube growth. The labelling from the JIM5 Ab (Fig. 7c) was intense and was found mainly around tip of the pollen tube; in contrast, the labelling of the 2F4 Ab was present in the form of small clusters (Fig. 7d). By light microscopy, we could not clearly confirm whether the observed labelling was present in the pollen tube wall or only in the ecm of nucellus. For this reason, the pectin composition of the pollen tube wall was studied in in vitro growing pollen tubes.

When mature archegonia were present in the ovule, considerable changes in the distribution pattern of low esterified and calcium cross-linked HG were observed in the nucellus. In comparison with the earlier developmental stage, the fluorescence intensity after labelling with JIM5 (Fig. 7c, f) and 2F4 (Fig. 7d, g) was clearly lower. In the ecm of the nucellar apex that did not contain pollen grains, the signals from both JIM5 (Fig. 7f) and 2F4 Abs (Fig. 7g) were distributed mainly as single fluorescence clusters of different sizes, and accumulation of esterified HG was also observed (Fig. 7e). At this time, the 2F4 Ab labelling was also present at the top of the archegonium over the neck cells (Fig. 7h). Our earlier investigations have demonstrated that HGs recognised by JIM7 and JIM5 were also localised in this area (Rafińska and Bednarska 2011).

Immunocytochemical localisation of HG in in vitro germinating pollen grains revealed the presence of only high-esterified HG (Fig. 7i). Accumulation of epitope recognised by the JIM7 Ab was observed in the intine around the site of pollen tube germination. In the pollen tube, labelling was present in the cell wall as well as in the cytoplasm, where it was visible in the form a small clusters. No low methyl-esterified or Ca^2+^-associated HGs were detected in the pollen grain or pollen tube (data not shown).

**Fig. 8:**
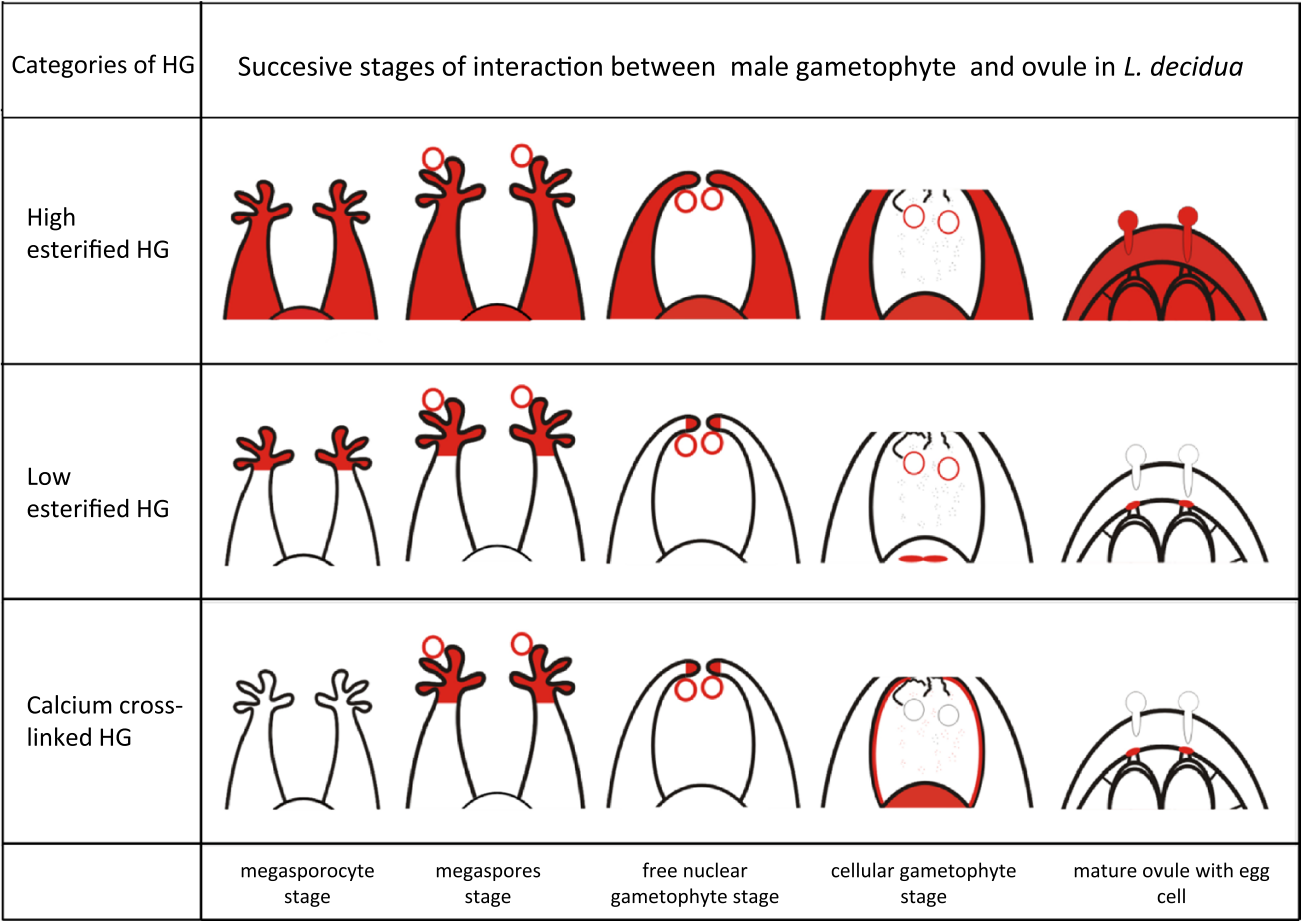
Schematic localisation of different categories of HG (*red colour*) in the male gametophyte and the ovule during successive stages of their interaction in *L. decidua*

Temporal and spatial changes in the distribution of the different categories of HG during male gametophyte–ovule interaction in *L. decidua* are summarised in Fig. 8.

## Discussion

### Pollen grain adhesion and its transfer to the micropylar canal

In the apoplast of the unpollinated stigmatic tip of *L. decidua* as well as in the stigmas of angiosperms (Bednarska et al. 2005; Sage et al. 2009), both high methyl-esterified and low methyl-esterified HG were localised. In this study, we showed that pollination induced changes in the pectin composition of the stigmatic tip, especially at the site of pollen grain adhesion where calcium cross-linked HG appeared. The presence of this category of HG at the site of physical contact between the ecm of the stigmatic tip and the pollen wall suggests that these molecules play an important role during the early interaction steps between the male gametophyte and the ovule in larch plants. Many reports have suggested that in angiosperms, low-esterified HG is responsible for the adhesion of the pollen grain to the stigma and that it most likely occurs by calcium cross-linking (Mollet et al. 2000). Here, we showed that in *L. decidua*, calcium cross-linked HG is involved in the adhesion between the pollen grain and stigmatic tip.

After a few days of pollination, the stigmatic tip and attached pollen grains were drawn into the micropylar canal. At this time, changes in the HG composition of the pollen grain cell wall were observed. The lack of calcium cross-linked HG indicates its degradation in the pollen wall during the pre-germinative period. Lysis of pectin is performed by polygalacturonase (Bonnin et al. 2002; Wolf et al. 2009). The accessibility of polygalacturonase to low-esterified HG, the substrate for this enzyme, requires disintegration of the ‘egg-box’ structure. Thus, it is probable that after entry of the pollen grains into the micropylar canal, dissociation of links between HG chains in the stigmatic tip cell as well as in the pollen wall occurs. We postulate that the disintegration of the ‘egg-box’ structure leads to the disappearance of adhesion between the male gametophyte and the stigmatic tip and, thus, allows the transfer of pollen grains to the nucellus.

In the pollen wall, significant levels of low methyl-esterified HG indicate the presence of large amounts of negatively charged COO-groups. It is known that their presence is associated with cell wall hydration and swelling (Zsivanovits et al. 2004). It is possible that swelling of low methyl-esterified HG, present in the pollen wall, causes exine rupture and shedding.

Moreover, ‘egg-box’ disintegration leads to the appearance of the free calcium ions in the micropylar canal. We propose that this pool of calcium ions can subsequently be bound by the HG, which is present in ECM of the micropylar canal. We have found that the presence of pollen in the micropylar canal induced changes in the pectin composition of its ecm. Calcium cross-linked HG appeared in the walls coating the micropylar canal and as single, irregular clusters in the canal secretion. This suggests that this category of HG is a component of the secretion that is necessary for the transfer of pollen grains to the nucellar apex.

### Pollen germination and pollen tube growth

Nucellar surface cells are secretory and degenerate during the transfer of pollen grains through the micropylar canal. At this time, the changes in the nucellus HG seemed to reflect the preparation of the environment for pollen germination and tube growth. During pollination, the nucellar cell walls showed only the presence of esterified HG. Our results suggest that when pollen was present in the micropylar canal, deesterification of HG and Ca^2+^ binding occurred in the ecm of the nucellus. Similarly, in angiosperm plants, pollination induced deesterification of HG present in the extracellular matrix of the style transmitting tissue (Lenartowska et al. 2001). A decrease in the level of deesterified and Ca^2+^-associated HG was observed in the nucellus during pollen grain germination and tube growth. Both these categories of HG were most likely degraded. Lysis of calcium cross-linked HG leads to Ca^2+^ release. Thus, we postulate that the precisely regulated degradation of this HG creates an optimal calcium environment for pollen grain germination and pollen tube growth. Many studies in both angiosperms and gymnosperms showed that Ca^2+^ plays a particularly important role in pollen grain germination and pollen tube growth (Malhó et al. 2000; Fernando et al. 2005; Lazzaro et al. 2005; Wu et al. 2008). Both lysis of deesterified HG (Lenartowska et al. 2001) and a strong increase in the Ca^2+^ level in the extracellular matrix of the pollinated solid style have been described previously in some angiosperms (Lenartowska et al. 1997). Thus, the mechanism of creating an optimal Ca^2+^ environment for pollen tube growth in the nucellus of the *L. decidua* ovule seems to be similar to that observed in the solid style of flowering plants. An additional aspect of HG lysis in larch plants is cell wall loosening, which facilitates penetration of this tissue by growing pollen tubes.

Characteristic accumulation of calcium cross-linked HG was visible above neck cells of the mature archegonium. Our recent investigations have shown that in this area, both high methyl-esterified HG and low methyl-esterified HG are also present (Rafińska and Bednarska 2011). The specific pectin composition of these cell walls indicates that this region could be involved in attracting and directing the pollen tube. In angiosperms, these functions are performed by the filiform apparatus of the synergids, where elevated levels of pectins were also observed (see the review by Li et al. 2009).

In the cell wall of germinating pollen grains high-esterified HG was still present at a low level. However, the lack of low methyl-esterified HG indicates that it undergoes lysis. At this time, high methyl-esterified HG was also present in the pollen cytoplasm, which most likely reflects its synthesis for the growing pollen tube. In gymnosperms, similar to angiosperms, HG is an essential component of the expanding pollen tube wall (Derksen et al. 1999; Fernando et al. 2005; Parre and Geitmann 2005; Chen et al. 2008; Wu et al. 2008; Dardelle et al. 2010; Lehner et al. 2010). In vivo, it was difficult to distinguish the wall of pollen tubes from the walls of the adjacent nucellar cells. At the border between the pollen tube and the nucellus, both low-esterified and calcium cross-linked HG were observed.

In the wall of *L. decidua* pollen tubes growing in vitro, only high methyl-esterified HG was localised, and no low methyl-esterified or Ca^2+^-associated HG was present. The punctate localisation of high methyl-esterified HG in the tube cytoplasm may indicate that its synthesis and secretion takes place during pollen tube growth. It should be noted that in vitro growing pollen tubes were very short, and the HG composition of their walls may not reflect the in vivo state. High methyl-esterified, but not deesterified, HG was detected in in vitro growing pollen tubes of *Pinus sylvestris* and *Pinus densiflora* (Derksen et al. 1999; Mogami et al. 1999). However, low methyl-esterified HG was detected in the walls of *Picea wilsonii*, *P. meyeri* and *P. bungeana* pollen tubes (Chen et al. 2008, 2009; Wu et al. 2008).

Low methyl-esterified HG is a main component of the angiosperm pollen tube wall. It is suggested that in the pollen tube wall, the negative charges of deesterified HG bind Ca^2+^, which imparts structural rigidity to the cell wall (Hepler and Winship 2010). Low methyl-esterified HG in pollen tubes of angiosperms is derived from successive deesterification of esterified HG originally deposited in the tip. The obtained results and literature data suggest that in the pollen tubes of some gymnosperms, this process does not occur (*L. decidua*, *P. sylvestris* and *P. densiflora*). It is possible that the shorter and much slower growing tubes of gymnosperms do not need mechanical support in the form of deesterified HG.

In summary, our investigations suggest that in the male gametophyte–ovule interaction in *L. decidua,* low methyl-esterified and calcium cross-linked HG play an important role (Fig. 8). HG was only deesterified in the cell walls and extracellular matrix of the ovule tissues that interact with male gametophyte, and a pool of low methyl-esterified HG was cross-linked by Ca^2+^. This last category of HG is most likely involved in adhesion between the pollen and the stigmatic tip and may be a reservoir of Ca^2+^ in ecm of the ovule. The precisely regulation of calcium cross-linked HG degradation might provide an optimal Ca^2+^ environment for pollen grain germination and pollen tube growth.

## Abbreviations

HG: Homogalacturonan (homopolymer of α-1,4-linked galacturonic acid)
PMEs: Pectinmethylesterases
RT: Room temperature
PG: Polyethylene glycol
PBS: Phosphate-buffered saline
BSA: Bovine serum albumin
DAPI: 4′,6-Diamidino-2-phenylindole
ecm: Extracellular matrix
